# Imaging Brown Adipose Tissue: Current State and Future Perspective

**DOI:** 10.7150/thno.111643

**Published:** 2025-08-16

**Authors:** Junhao Li, Li Wang, Shiyao Wu, Guifen Yang, Long Jiang Zhang

**Affiliations:** 1 Department of Radiology, Jinling Hospital, The First School of Clinical Medicine, Southern Medical University, Nanjing, 210002, China.; 2 Department of Radiology, Jinling Hospital, Affiliated Hospital of Medical School, Nanjing University, Nanjing, 210002, China.; 3 Department of Nuclear Medicine, Jinling Hospital, Affiliated Hospital of Medical School, Nanjing University, Nanjing, 210002, China.

**Keywords:** brown adipose tissue, imaging, physiology, function, metabolism.

## Abstract

Brown adipose tissue (BAT) is a specialized type of fat tissue that utilizes various nutrients and is considered a novel therapeutic target for metabolic, disorders, cardiovascular diseases, and certain types of cancer. However, the current standard imaging method for BAT, ^18^F-flurodeoxyglucose positron emission tomography computed tomography ([^18^F] FDG PET-CT), fails to meet clinical demands due to its prohibitive costs, prolonged imaging times, and radiation exposure, which are significant concerns for longitudinal studies. To overcome these limitations, emerging imaging modalities are being explored, aiming to address these challenges by focusing on alternative biomarkers of BAT, such as lipid content, perfusion, density, thermal emissions, and mitochondrial activity. Advanced imaging methods have been developed for precise imaging, facile operation, and broad applicability. In this review, we provide a brief overview of BAT physiology and function, as well as current advancements in BAT imaging methods, including positron emission tomography, single photon emission computed tomography, magnetic resonance imaging, computed tomography, infrared thermography, optoacoustic imaging, and Xenon-enhanced imaging. Future perspectives, such as the application of artificial intelligence to BAT imaging, are also discussed.

## Introduction

Brown adipose tissue (BAT) is a thermogenic fat depot that dissipates energy as heat by uncoupling oxidative phosphorylation, playing a key role in energy expenditure and thermoregulation 1-3. Recent studies have revealed that BAT does far more than just thermogenesis, serving a positive role in metabolism, the endocrine system, and immunity. BAT consumes substantial nutrients, including free fatty acids, during heat production, thereby reducing serum triglyceride levels and improving metabolic profiles 4-6. Furthermore, a novel mechanism, branched-chain amino acids (BCAA) - nitrogen flux in BAT, has recently been identified to improve systemic glucose homeostasis independently of thermogenesis 7, 8. Moreover, BAT secretes adipokines through endocrine pathways to crosstalk with other organs 9-13. For example, BAT secretes 3-methyl-2-oxovaleric acid (MOVA), 5-oxoproline, and β-hydroxyisobutyric acid (BHIBA) to increase energy expenditure in skeletal muscle 11, 12,13-dihydroxy-9Z-octadecenoic acid (12,13-diHOME) to protect the cardiovascular system 12, and fibroblast growth factor 21 (FGF21) to improve insulin secretion 13. Emerging evidence further implicates BAT in immune-related functions, such as regulating liver immune cell infiltration and antagonizing inflammation 14. Notably, BAT is regarded as a unique niche for islet organoid transplantation 15, 16.

Given these multifaceted benefits, BAT-focused clinical studies warrant further exploration, with imaging playing a pivotal role in this context. To date, the most commonly used method for BAT imaging is ^18^F-Fluorodeoxyglucose ([^18^F] FDG) positron emission tomography (PET), which is considered the gold standard for detecting BAT. Despite its widespread clinical adoption, this approach faces inherent limitations, including high costs, demanding preparation protocols (which include cold exposure requirements), radiation exposure concerns, and the lack of clinical indications for BAT imaging. These limitations underscore the urgent need for clinically translatable, accessible, and precise BAT imaging technologies.

Recent advances in imaging techniques and artificial intelligence (AI) are accelerating the development of next-generation BAT assessment methods. This review first outlines the physiology and function of BAT, followed by a comprehensive analysis of advanced BAT imaging modalities, including PET, single photon emission computed tomography (SPECT), magnetic resonance imaging (MRI), computed tomography (CT), infrared thermography (IRT), optoacoustic imaging, and Xenon-enhanced imaging. We summarize these methods and list their advantages and limitations in **Table 1**. Critically, we highlight breakthrough technologies such as [^18^F] AraG, chemical exchange saturation transfer (CEST) imaging, and AI-based approaches, discussing their potential for significant clinical impact. To the best of our knowledge, this represents the first review specifically introducing AI applications in BAT imaging, aiming to assess its potential to detect BAT and resolving current technical challenges. Finally, we discuss key knowledge gaps and future directions to accelerate the translation of BAT imaging from research tools to clinical practice.

## BAT physiology

**Figure 1:**
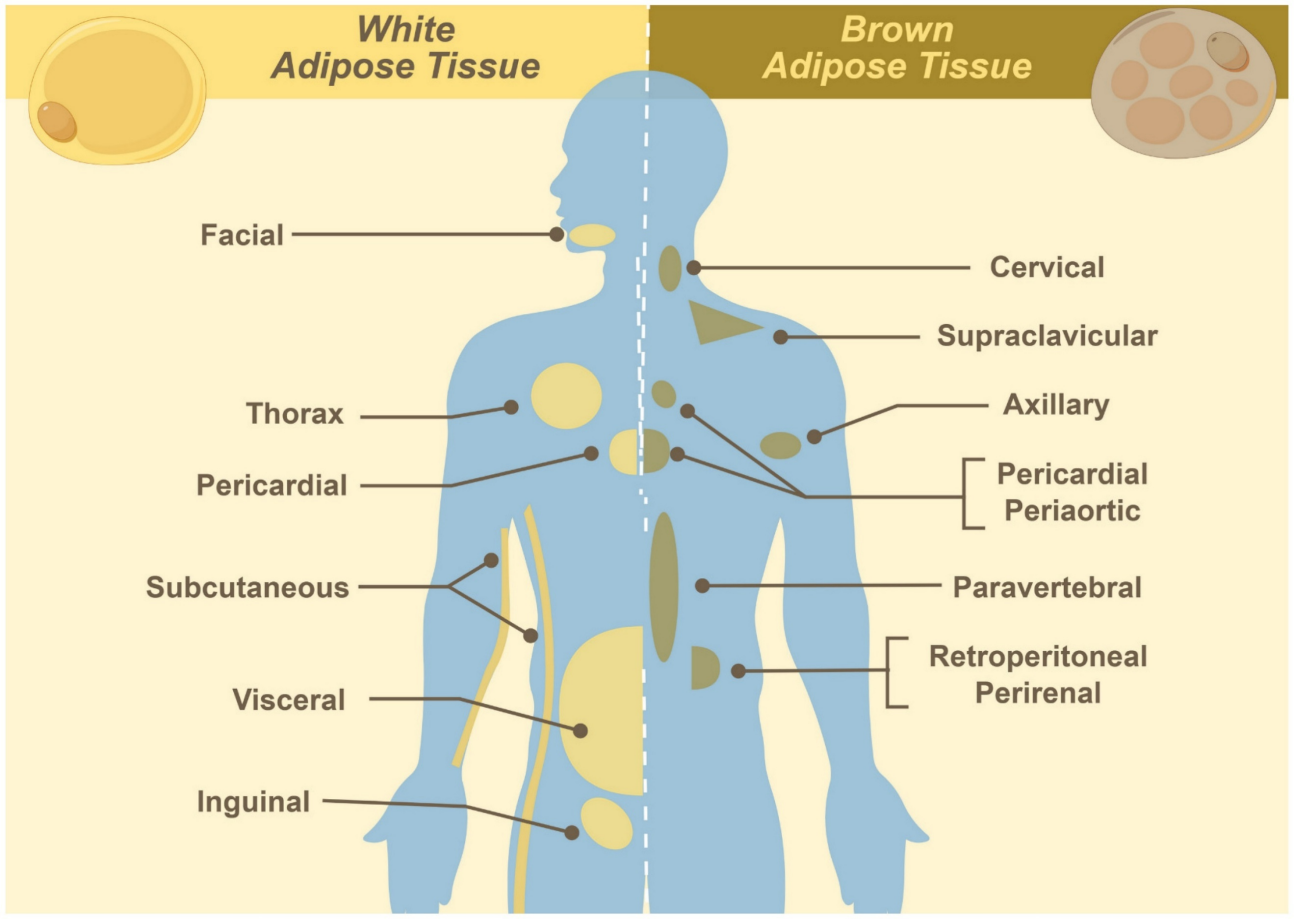
Distribution of white and brown adipose tissues.

BAT is a specialized type of fat tissue characterized by its brown appearance, which is due to the presence of abundant mitochondria. It originates from mesodermal precursors during mid-to-late gestation, reaching its peak in metabolic activity at birth, and progressively declining with age. In neonates, BAT is widely distributed in cervical, supraclavicular, axillary, mediastinal, paraspinal, and periadrenal regions (**Figure 1**). During adulthood, BAT becomes functionally quiescent and quantitatively reduced, persisting primarily in the cervical to supraclavicular depot, along the paravertebral axis, and adjacent to major vasculature. Quantitatively, BAT constitutes approximately 50 - 500 grams (0.2 - 3.0% of total adipose mass) in individuals aged 20 - 50 years, showing significant correlations with sex, age, and body mass index 1, 3. The primary function of BAT is non-shivering thermogenesis, consuming substantial nutrients via dedicated plasma membrane transporters to maintain body temperature 1-3. Substrate uptake (e.g., glucose and free fatty acids) forms the basis of PET imaging, where tracer concentration correlates with BAT activation 17-20**.**

Histologically, BAT composition resembles that of white adipose tissue (WAT), consisting of adipocytes, precursor cells, fibroblasts, and immune cells. The critical distinction lies in adipocyte types: Different from WAT, BAT comprises multilocular adipocytes with distinct gene expression profiles. These cells feature central nuclei surrounded by numerous small lipid droplets. This unique morphology reduces fat content and increases tissue density, forming the basis for Dixon MRI and CT imaging 21, 22. Additionally, brown adipocytes contain an abundance of mitochondria 1-3, providing targets for mitochondrial metabolic tracers 23, 24 and T2*-weighted imaging (exploiting rapid signal decay) 25. In addition, BAT exhibits denser capillary and nerve fiber networks. During non-shivering thermogenesis activation, generated heat dissipates throughout the body via this capillary network 26. The high vascular density also enables perfusion-based imaging (e.g., blood oxygenation level-dependent (BOLD) MR 27, optoacoustic imaging 28) and enhanced imaging (e.g., Xenon-enhanced imaging 29).

**Figure 2:**
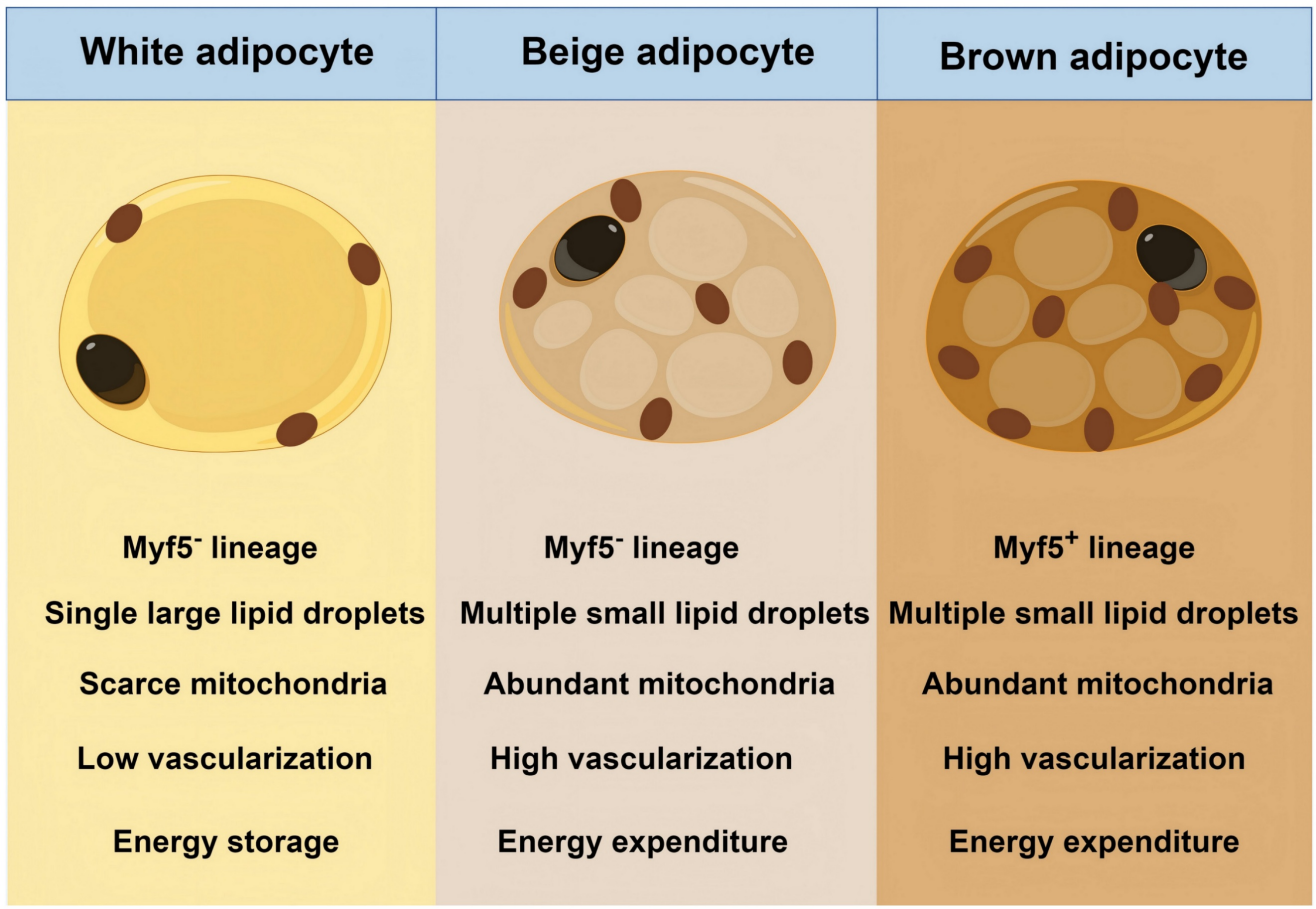
** Differences between the three types of adipocytes.** Myf5: myogenic transcription factor 5.

Beyond classical BAT and WAT, a third type, beige adipose tissue, has been discovered in WAT depots. Beige adipose tissue emerges in WAT depots during cold exposure or β-adrenergic stimulation through a reversible browning process. Beige adipocytes share structural similarities with brown adipocytes, including multilocular lipid droplets and high mitochondrial content 9, 30. However, they originate from myogenic transcription factor 5 (Myf5)-negative mesodermal stem cells, which are identical to white adipocytes but contrast with the Myf5-positive origin of classical brown adipocytes 9, 30. Despite distinct progenitor lineages, beige adipocyte acts in a thermogenic role. **Figure 2** presents the difference between BAT, beige adipose tissue, and WAT. Herein, we classify beige adipose tissue as BAT due to their functional and imaging similarities.

The mechanism of non-shivering thermogenesis in BAT has been progressively elucidated. Uncoupling protein 1 (UCP1)-dependent thermogenic mechanism represents an early-characterized pathway 2. This classical pathway involves UCP1, a BAT-specific mitochondrial inner membrane protein, which, upon β-adrenergic stimulation, acts as a proton channel, to dissipate the electrochemical gradient, uncoupling mitochondrial respiration from ATP synthesis and generating heat 31. As UCP1 function is conserved between humans and rodents 32, it remains the most studied BAT thermogenic pathway.

**Figure 3:**
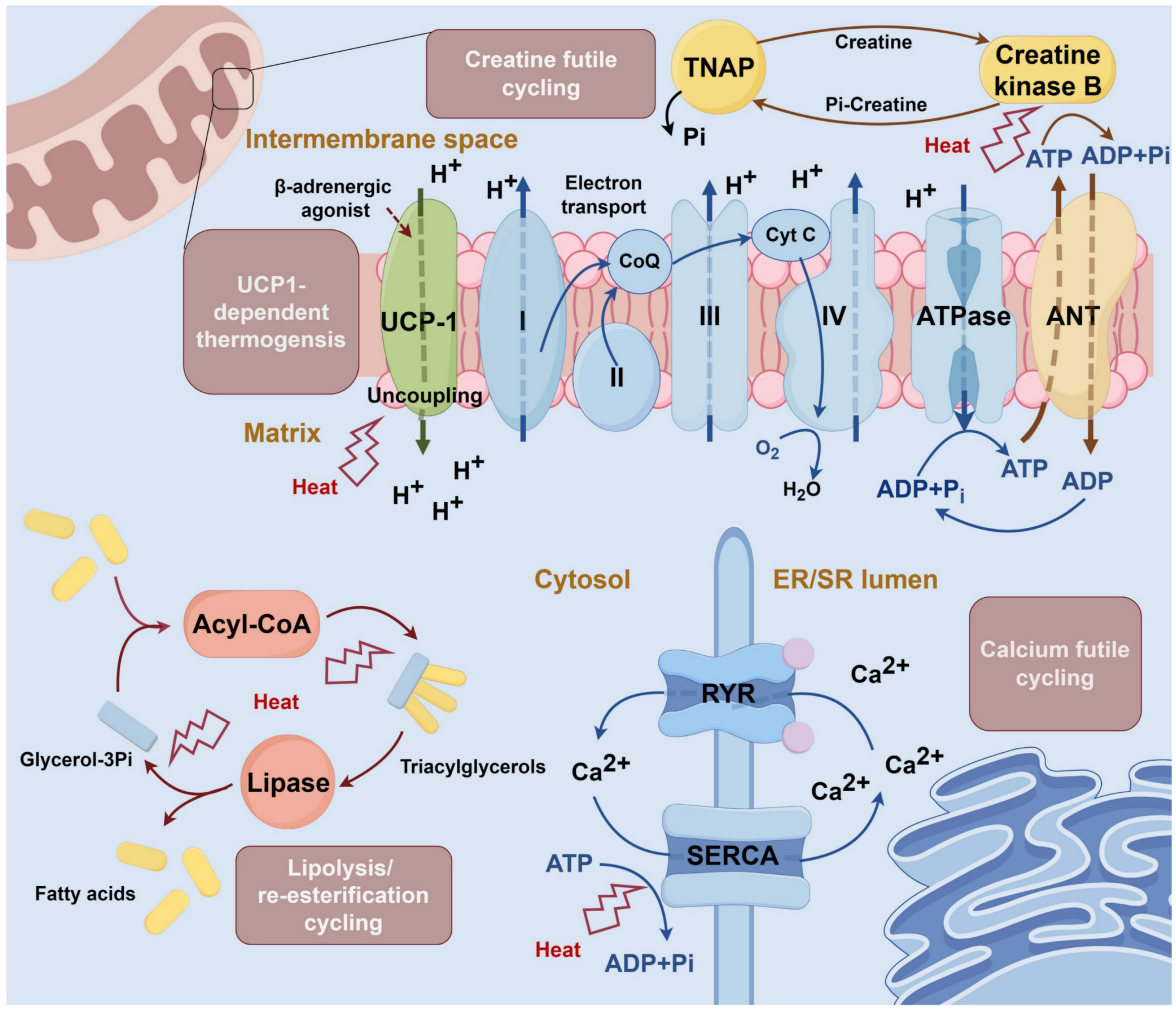
** Mechanism of thermogenesis in brown adipose tissue.** Uncoupling protein 1 (UCP1)-dependent thermogenesis: UCP1 is activated by β-adrenergic agonists and leads to the opening of the hydrogen ion channel. The channel dissipates the electrochemical gradient and uncouples mitochondrial respiration from ATP synthesis, generating heat 31. Creatine cycling: In the mitochondrial intermembrane space, creatine is phosphorylated to phosphocreatine by creatine kinase B with the consumption of ATP. Phosphocreatine is then hydrolyzed by tissue-nonspecific alkaline phosphatase (TNAP) and continues the loop 34. Calcium cycling: Calcium ions are transferred into the endoplasmic reticulum (ER) by sarcoendoplasmic reticulum calcium ATPase (SERCA) and then released through ryanodine receptor (RYR). Cycling continuously consumes ATP 35. Glycerolipid cycling: The lipolysis and esterification of glyceride are continuously processed in the cytosol and consume ATP 36. ANT: adenine nucleotide translocator; ER/SR: endoplasmic reticulum / sarcoplasmic reticulum; RYR: ryanodine receptor; SERCA: sarcoendoplasmic reticulum calcium ATPase; TNAP: tissue-nonspecific alkaline phosphatase; UCP1: uncoupling protein 1.

Continued in-depth studies have revealed three additional UCP1-independent thermogenic mechanisms 31, 33-36: 1) Creatine cycling: Mitochondrial creatine kinase B phosphorylates creatine using ATP, followed by tissue-nonspecific alkaline phosphatase (TNAP)-mediated hydrolysis, establishing a futile cycle that dissipates energy as heat 34, 37; 2) Calcium cycling: Sarcoendoplasmic reticulum calcium ATPase (SERCA) transports calcium ions into the endoplasmic reticulum (ER) using ATP, with subsequent release via ryanodine receptor (RYR) 35; 3) Glycerolipid / free fatty acid cycling: Continuous glycerolipid hydrolysis and re-esterification, consuming ATP in the process 36. The summary of these thermogenic mechanisms is shown in **Figure 3**. The additional thermogenic mechanisms offer potential imaging targets, as demonstrated by creatine-based CEST, which detects BAT activity through changes in creatine concentration 38. Conversely, calcium- and glycerolipid-based imaging has received limited investigation due to low specificity.

### BAT function

For decades, BAT was considered functionally relevant only in infants and children, with presumed minimal presence in adults. This perspective was overturned in the early 2000s when BAT was identified in cold-exposed adults 17-19, sparking renewed interest in its potential function beyond thermoregulation.

BAT has emerged as a therapeutic target for obesity and metabolic disorders due to its energy-expending capacity, aligning with anti-obesity strategies. This hypothesis is supported by the higher prevalence of BAT in lean versus obese individuals. However, quantitative analyses indicate BAT's energy expenditure ranged from 27 - 123 kcal per day at room temperature to 211 kcal per day during cold exposure 39, insufficient for clinically meaningful weight loss (requiring at least 500 kcal per day). Besides, despite BAT exhibiting 2- to 8-fold higher glucose uptake rate than skeletal muscles, its limited mass (~0.2% of muscle mass) contributes only 1% of total body glucose utilization 40-42. Longitudinal trials demonstrated no weight loss after 4 - 6 weeks of cold exposure 43, 44, or β-adrenergic agonist treatment 45. Additionally, BAT failed to mediate metabolic adaptation in overfed non-obese participants during 8-week interventions 46. However, murine studies have shown weight loss, increased energy expenditure, and improved insulin resistance following BAT activation or transplantation 47, which is potentially attributable to the proportionally larger BAT volume in mice. Besides, transplanted BAT undergoes whitening, a process that would be aggravated in obese hosts, indicating susceptibility to, rather than modification of, the host metabolic environment 48-50. Despite the limited anti-obesity efficacy of BAT, it may facilitate weight maintenance 51, warranting further investigation.

Beyond thermogenesis, BAT functions as an endocrine organ 10, 52, secreting adipokines that regulate systemic insulin sensitivity, glucose homeostasis 53-55, and cardiovascular health 12, 56. Although weight-loss effects remain limited, metabolic benefits are well-documented. A 10-day cold acclimation increased peripheral insulin sensitivity by ~43% in type 2 diabetes patients (n = 8) 57, while 4-week β_3_-agonist (mirabegron) treatment improved glucose effectiveness by 34% and insulin sensitivity by 36% 45. Cardioprotective effects were also consistently demonstrated in mice, including the selective uptake of fatty acidsfrom triglyceride-rich lipoproteins, reduced plasma triglyceride and cholesterol levels, and inhibited atherosclerosis 4. Other mouse experiments have shown that BAT-derived neuregulin-4 mitigated vascular inflammation and remodeling 56, 58. BAT also secretes a series of regulatory factors that target distant organs, such as the heart, pancreas, and skeletal muscle, to regulate systemic metabolism and cardiovascular health 10. A few preliminary clinical studies have demonstrated that BAT is correlated with lower coronary artery calcification scores 59, reduced cardiovascular events 60, and lower prevalence of cardiometabolic diseases (particularly in obesity) 61. Prospective studies in high-risk cohorts (e.g., diabetes and cardiovascular disease cohorts) are required to validate the clinical importance of BAT's endocrine function.

Except for endocrine function, emerging evidence highlights BAT as a transplantation site for pancreatic islets in type 1 diabetes. Islets transplanted into the supraclavicular BAT depot exhibit significantly delayed autoimmune and allograft rejection compared to kidney capsule grafts, while maintaining viability and endocrine function 15, 16, 62. This protective effect is likely attributable to BAT's unique microenvironment. Dense vascular and neuronal networks ensure nutrient supply 1-3, and enriched anti-inflammatory macrophages and T cells mitigate immune-mediated damage 63-65. Procedural accessibility further supports BAT as a potential ideal site for islet transplantation. Moreover, cold-activated BAT depletes blood glucose, impairing glycolysis-dependent metabolism in cancer cells 66. This metabolic competition mechanism has inspired the use of UCP1-engineered adipocyte transplantation to create a nutrient-deprived microenvironment that suppresses tumor growth 67. Through its intrinsic metabolic activity, BAT establishes a novel metabolic approach to cancer therapy.

## BAT imaging

### PET

PET has long been the imaging gold standard for BAT diagnosis. As an energy-consuming tissue, BAT volume and activity can be reflected by PET imaging through metabolic energy expenditure. PET has the advantage of displaying specific substance metabolism. Based on the physiological mechanism of brown fat, tracers targeting glucose, lipids, and mitochondria have been developed and have demonstrated good imaging. Current research on BAT mainly employs PET imaging, providing insights into BAT physiological characteristics, activation pathways, and systemic metabolic effects.

#### Glucose

Glucose serves as a key metabolic substrate for BAT and the molecular target of [^18^F] FDG, establishing [^18^F] FDG PET as the current gold-standard method for BAT detection *in vivo*. Initial studies in 2009 confirmed the presence BAT in adult males using [^18^F] FDG PET-CT 17-19. Subsequently, [^18^F] FDG has been employed to quantify BAT volume and activity, identify its determinants, monitor WAT browning, and evaluate the efficacy of activation strategy 68. However, [^18^F] FDG reflects glucose metabolism rather than BAT volume, imaging only the active part of BAT. Consequently, [^18^F] FDG-based BAT imaging requires stimulation (e.g., cold exposure or drug treatment) and is confounded by clinical variables including body mass index, age, sex, and glycemia 69.

To address these challenges, a standardized imaging workflow has been developed. In 2016, Chen et al. established the Brown Adipose Reporting Criteria in Imaging STudies (BARCIST 1.0) to enhance study comparability and reproducibility. In this guideline, BAT is defined as the standardized uptake value based on lean body mass ≥ 1.2 on PET images, and -190 to -10 Hounsfield Units (HU) on CT, excluding obvious non-fat tissues within this range, such as lungs 70. The guideline also standardizes preparation protocols, BAT activation procedures, and imaging parameters, solidifying PET-CT's status as the gold standard for BAT imaging 70.

To standardize the research, most clinical trials for BAT are based on [^18^F] FDG imaging. However, there are several limitations for [^18^F] FDG imaging: high costs, complex imaging protocols, and unavoidable radionuclide radiation, which collectively hinder longitudinal and large-scale population studies. Moreover, [^18^F] FDG may not reliably quantify BAT activity. For instance, insulin-resistant patients present reduced [^18^F] FDG uptake in BAT despite normal fatty acid uptake and oxygen consumption, indicating preserved BAT function 70. Similarly, murine studies revealed preserved [^18^F] FDG uptake in defective thermogenic BAT following drug stimulation, suggesting discordance between [^18^F] FDG uptake and functional activity 71, 72.

#### Fatty acids

Fatty acids serve as the primary fuel for BAT thermogenesis, positioning them as theoretically ideal imaging targets. However, the clinical translation of fatty acid-based BAT imaging remains limited, partially because these tracers were initially developed for myocardial function imaging in the late 1980s and have remained in preliminary research stages for BAT imaging, with optimal detection methods and tracer dosage still under exploration 20, 73. Moreover, the short half-life of specific radiolabeled fatty acids (e.g., 20 min for [^11^C] palmitate) further restricts their clinical applicability 20. These limitations pose significant challenges for the development and clinical application of fatty acid-labeled radiotracers.

Currently, the most commonly used fatty acid tracers in human studies include 14(R,S)-[^18^F] Fluoro-6-thia-heptadecanoic acid ([^18^F] FTHA) and [^11^C] palmitate. [^18^F] FTHA uptake quantifies BAT activity, demonstrating increased fatty acid uptake following cold exposure 42. Notably, Blondin et al. revealed that fatty acid tracer imaging was unaffected in diabetes, potentially providing a more accurate reflection of activated BAT than glucose tracers 74. Murine studies using [^18^F] FTHA revealed a distinct pattern of lipid metabolism, characterized by a rapid increase in uptake during acute (2 h) and chronic (6 h) cold exposure, followed by a gradual decline after withdrawal of stimulation. These studies also demonstrated the pattern of oxidative activity and glucose uptake, as measured by [^11^C] acetate and [^18^F] FDG, respectively, which increased significantly only after chronic cold stimulation and decreased rapidly upon rewarming. These findings suggest that BAT prioritizes the utilization of fatty acids for thermogenesis.

To further investigate this mechanism, researchers employed a lipolysis inhibitor to elucidate the role of lipolysis in BAT thermogenesis. This intervention resulted in marked suppression of BAT activation, including oxidative activity and glucose and fatty acid uptakes, confirming the significant role of lipolysis in maintaining BAT thermogenesis 75. Despite these advances, [^18^F] FTHA uptake rates in BAT were substantially lower than [^18^F] FDG (2.3 ± 0.8 μmol/min vs. 10.8 ± 4.5 μmol/min), attributed to preferential consumption of intracellular lipid stores over circulating fatty acids 76. Additionally, free fatty acid uptake is nonspecific, leading to off-target accumulation in the liver and intestines 55. Several novel fatty acid tracers, including [^18^F] monoacylglycerol lipase, [^18^F] oleic acid analog, and [^123/125^I] β-methyl-ρ-iodophenyl-pentadecanoic acid (BMIPP), have shown improved specificity and kinetics in preclinical studies 77-79.

Together, these studies highlight the advancements in fatty acid-labeled PET imaging for BAT detection, indicating that future research should focus on optimizing tracer specificity, uptake kinetics, and clinical translation. Given their myocardial origins, fatty acid tracers may also help elucidate BAT's relationship with cardiovascular diseases.

#### Mitochondria

The [^18^F] arabinosyl guanine analog ([^18^F] AraG) is a novel mitochondrial metabolic tracer initially designed to track activated T cells through mitochondrial DNA synthesis, providing a non-invasive method for assessing system-wide immune status 80. Recent studies revealed that [^18^F] AraG could reflect activated BAT due to its high mitochondrial content 23. In terms of imaging mechanism, [^18^F] AraG, as a nucleoside analog, participates in mitochondrial DNA synthesis 81. During stimulation, mitochondrial biogenesis increases dramatically in both T cells and BAT cells, resulting in a high uptake of [^18^F] AraG 82. This dual-targeting capability enables simultaneous assessment of BAT activity and systemic immune function, offering unique insights into their interaction. Notably, one study identified a potential link among the immune system, the nervous system, and adipose tissue. Specifically, BAT activation was found to coincide with neuroinflammation, alongside suppressed immune activity in lymph nodes. These observations suggest that neuroinflammation may trigger BAT activation, which subsequently regulates the immune response to mitigate immunological damage 23. Additionally, [^18^F] AraG was also applied in monitoring immune response to cancer immunotherapy and chemotherapy 82-84, opening new areas for investigating BAT function in oncology. While BAT has been shown to suppress tumor growth via metabolic competition 66, other studies have reported that cachexia-induced adipose browning through type 2 immunity may exacerbate the dissipation of energy 85. [^18^F] AraG could help reconcile this paradox by dynamically monitoring BAT-immune crosstalk during cancer progression, shedding light on BAT's dual role in oncology.

Another promising BAT imaging target is the translocator protein (TSPO), a mitochondrial outer membrane protein involved in cholesterol transport and steroid hormone synthesis 86. Although TSPO tracers were initially developed for neurological and psychiatric disorders 87, they can effectively visualize BAT due to its high mitochondrial density. Notably, TSPO imaging captures total BAT volume, including inactive depots, making it independent of metabolic activity. This feature makes TSPO imaging particularly valuable for mapping the distribution of BAT. Several TSPO PET tracers, including N-(2-(2-[^18^F] Fluoroethoxy) benzyl)-N-(4-phenoxypyridin-3-yl) acetamide ([^18^F] FM-PBR28), N-[2-[2-[^18^F] Fluoroethoxy] benzyl]-N-(4-phenoxypyridin-3-yl) acetamide ([^18^F] FEPPA), and 7-(2-[^18^F] Fluoroethyl)-6,8-dihydro-5H-pyrido [2,3-b] indole ([^18^F] FDPA), have shown promise in preclinical studies, successfully identifying BAT and even newly formed beige fat without stimulation 24, 88, 89.

### SPECT

SPECT offers a nuclear imaging alternative with lower radiation exposure and cost than PET, thereby enhancing its accessibility for larger populations. However, SPECT's spatial resolution and sensitivity are inherently limited by its photon acquisition method, resulting in prolonged acquisition time (typically 15 min per bed position) 90. Moreover, quantitative accuracy is compromised by depth-dependent non-uniform attenuation of single photons and uncontrollable factors (including dead-time effect and spill-over effect), restricting cross-study comparability 91. Despite these limitations, SPECT has proven feasible for BAT imaging by targeting its rich vascularization and sympathetic innervation. Specifically, Wanda et al. successfully mapped BAT sympathetic activity using [^123^I] metaiodobenzylguanidine ([^123^I] MIBG), a sympathetic nerve tracer 92, while Aarondee et al. demonstrated increased blood perfusion with [^99m^Tc] methoxyisobutylisonitrile ([^99m^Tc] MIBI) 93. Notably, Zhang et al. found distinct metabolic patterns between beige adipose depots and classical BAT depots using [^123/125^I] β-Methyl-*p*-iodophenyl-pentadecanoic acid ([^123/125^I] BMIPP). Specifically, [^123/125^I] BMIPP additionally visualized classical and anterior subcutaneous WAT depots (beige adipogenesis sites), which were typically undetectable with glucose-based imaging. Conversely, [^123/125^I] BMIPP showed no significant uptake in the posterior cervical region (classical BAT depots), whereas [^18^F] FDG PET-CT did 79. These findings revealed distinct substrate preferences across BAT and beige depots, highlighting metabolic heterogeneity in adipose tissue. Besides, Frankl et al. showed that [^123/125^I] BMIPP was more closely related to the fold-change in metabolic rate than [^18^F] FDG PET-CT 94, indicating that [^123/125^I] BMIPP may be a promising tool for beige adipocyte investigation.

### MRI

MRI offers superior resolution for soft tissues. Its diverse imaging sequences enable the detection of subtle differences between brown fat and white fat. Additionally, MRI possesses inherent advantages of being non-invasive and radiation-free, making it one of the optimal imaging methods for long-term monitoring of BAT. Currently, specific MRI sequences have been developed targeting differences in fat-water ratio, creatine concentration, and blood perfusion between brown and white fat, yielding favorable imaging outcomes.

#### Dixon sequence

The Dixon sequence, also termed fat-water imaging, enables BAT detection by quantifying tissue-specific fat fractions through differential magnetic responses. In the context of BAT, where mitochondria-rich adipocytes exhibit lower lipid content than white fat, this technique provides a distinction between the two types of adipose tissues 21. Studies using Dixon sequences have demonstrated that the BAT fat fraction decreases during cold exposure, reflecting the consumption of intracellular lipid droplets during thermogenic activation 95, 96. However, detecting BAT by Dixon sequences still faces challenges. For instance, Dixon is prone to magnetic field inhomogeneity artifacts and phase wrapping. To address these limitations, modified techniques, like iterative decomposition of water and fat with echo asymmetry and least-squares estimation (IDEAL) and fat fraction-based Z-spectrum imaging, have been introduced 97, 98. A more significant challenge arises from the overlapping fat fraction values between BAT and WAT. Specifically, Jones et al. reported considerable variation in BAT fat fraction among individuals, as well as temporal fluctuations within the same subject. The variable fat fraction complicates the establishment of an optimal cut-off value to distinguish BAT from WAT 21. Consequently, these factors currently limit the diagnostic utility of the technique, despite its physiological relevance.

#### T2* weighted imaging

T2* mapping offers a promising approach for BAT imaging due to its unique magnetic properties. The abundant mitochondria in BAT adipocytes, which contain iron-rich inner mitochondrial membranes and water-fat interface, accelerate signal decay in BAT compared to WAT, resulting in lower T2* values 25. Ouwerkerk et al. established a T2 cut-off value of 76 milliseconds for distinguishing BAT from WAT, achieving a sensitivity of 85% and a specificity of 95% 99. Furthermore, other studies showed that T2* imaging can monitor cold-induced thermogenesis 100. Nevertheless, BAT activation introduces confounding variables, including increased oxygen consumption, blood flow, and local-tissue temperature alterations, which may lead to uncertain changes in T2* value and potentially compromise measurement accuracy 101. Consequently, this physiological complexity limits the reliability of the technique for quantitative assessments of active BAT.

#### CEST

**Figure 4:**
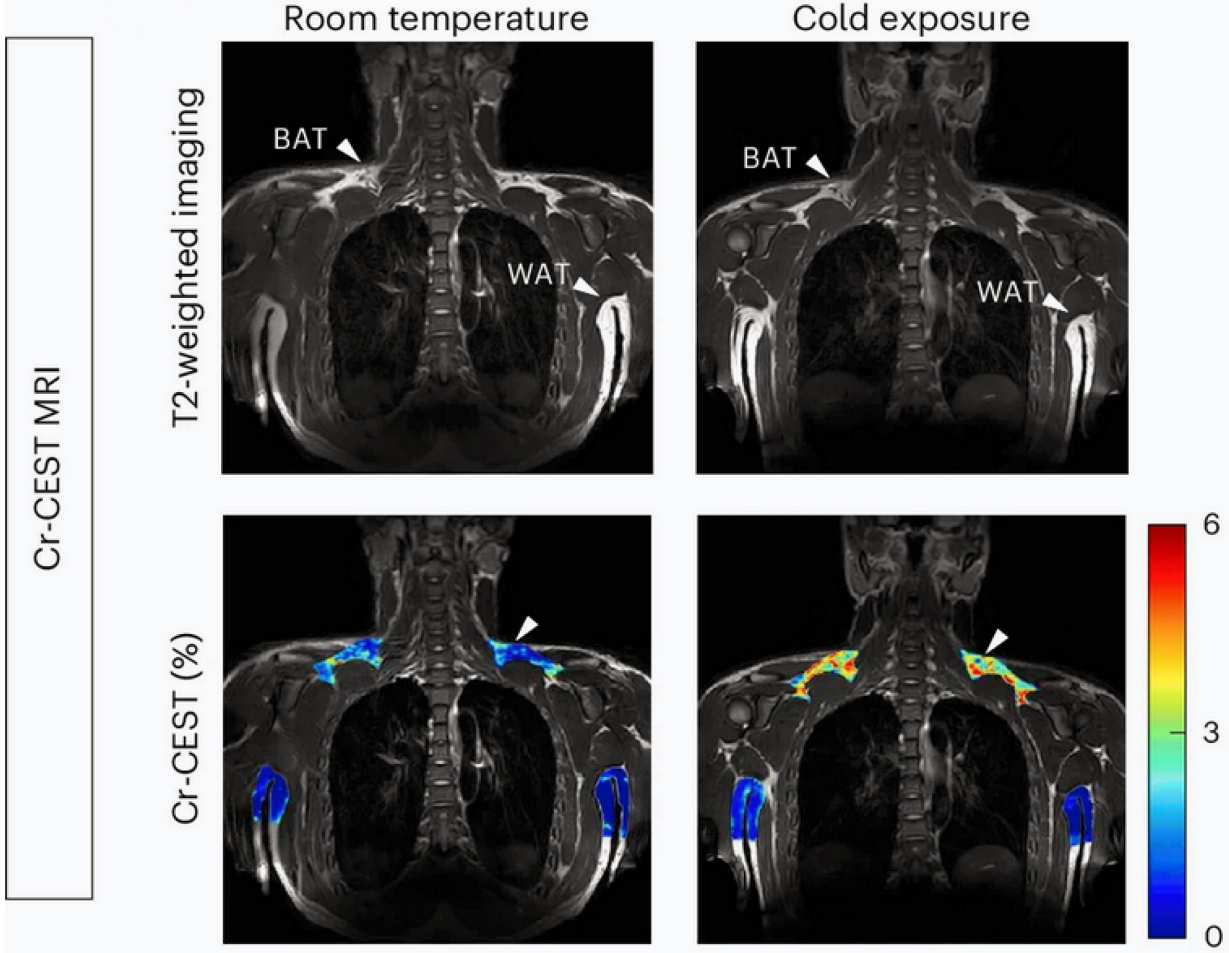
** Creatine-based CEST imaging of brown adipose tissue.** Brown adipose tissue (BAT) and white adipose tissue (WAT) presented in T2-weighted imaging (upper plane) and creatine-based chemical exchange saturation transfer (CEST) imaging (lower plane). BAT is reflected at room temperature (lower plane, left, white arrow), while the signal of BAT is increased after cold exposure (lower plane, right, white arrow). Figure 4 is reproduced with permission from Springer Nature (Nat Metab. 2024; 6: 1367-79), an open access article under a CC-BY 4.0 license. BAT: brown adipose tissue; CEST: chemical exchange saturation transfer; MRI: magnetic resonance imaging; WAT: white adipose tissue.

Recently, a novel metabolic MRI sequence, CEST, was applied to *in vivo* visualize BAT 102. CEST imaging detects metabolites by exploiting the exchange of protons between molecules and bulk water. When saturation is applied at the resonant frequency of specific metabolite protons, the effect is transferred to water protons, thereby reducing the bulk water signal. This measurable signal change reflects the concentration of the targeted protons 103, 104. Notably, BAT exhibits a reduced fat-water fraction and elevated amide proton transfer, which are correlated with BAT activity. Based on this mechanism, Cat et al. quantified the mass and activity of BAT with fat-water-fraction and amide proton transfer measurements from CEST 38, 102. Their study successfully demonstrated the dynamic changes in fat-water fraction and protein concentrations in BAT during the drug-stimulated process in mice 102. Moreover, Cai et al. targeted creatine to reflect BAT activity because the futile creatine cycle represents one mechanism of BAT thermogenesis 31, 33, 34. They validated the feasibility of the method using [^18^F] FDG PET scans and thermogenesis-related gene expression as the reference standard. Remarkably, the results showed that creatine-based CEST was more sensitive to BAT activity than [^18^F] FDG PET, detecting BAT even at room temperature (**Figure 4**) 38.

Cai et al. further investigated the impact of physical factors on creatine signal, including temperature, pH, and direct water saturation, which showed little effect on BAT imaging 38. However, as futile creatine cycling in parallel with UCP1-imediated thermogenesis, creatine-CEST may not fully capture UCP1-associated thermogenic dysfunction. Thus, its ability to reliably reflect BAT activation in metabolically impaired subjects remains to be further demonstrated 105. Further studies should be performed in exceptional cases, such as UCP1-deficient mice or diabetic patients, to clarify the performance of creatine-CEST. Aside from a few unresolved issues, this endogenous metabolic MRI technique requires no tracers or contrast agents, no cold stimulation and no radiation exposure, holding a promise for future clinical applications.

#### BOLD imaging

Functional MRI is paving the way for dynamic assessments of BAT activity, enabling non-invasive evaluation of BAT blood flow and metabolic changes induced by cold or drug stimulation. Chen et al. demonstrated the feasibility of BOLD for assessing BAT activity by detecting characteristic patterns of oxygen consumption and perfusion that reflect BAT thermogenesis 27. Further, Panagia et al. established the clinical relevance of this technique by revealing impaired BAT volume and function in heart failure models. These parameters showed a strong correlation with myocardial perfusion, indicating potential cardio-metabolic crosstalk 106. Collectively, these advances position BOLD as a valuable platform for studying the thermogenic function of BAT and its potential implications for metabolic health.

MRI is considered a leading modality for non-invasive BAT assessment, combining high soft-tissue contrast, radiation-free imaging, and versatility in imaging sequences. This technique offers diverse imaging sequences that exploit subtle features of BAT, mainly based on water content, blood perfusion, and specific metabolites. MRI can map the BAT anatomical volume (e.g., Dixon and T2* weight imaging) and its functional activity (e.g., creatine-CEST and BOLD sequences). Although it may slightly underestimate BAT volume, MRI enables BAT quantification without cold exposure, offering potential clinical applications for population-scale studies. Capitalizing on its repeatability, MRI captures distinct physiological dynamics, including metabolic activity peaks within hours following physiological stimulation (typically reaching steady state), while BAT recruitment occurs over weeks 2. Thus, MRI serves as a sensitive biomarker for monitoring BAT activation and its links to metabolic disorders, cardiovascular risks, and oncological processes. Additionally, MRI has become essential for evaluating BAT activation strategies, including cold exposure regimens and pharmacologic interventions targeting adrenergic pathways. Multimodal MRI further expands capabilities by integrating depot mapping through combined anatomical imaging (e.g., Dixon MRI) and functional imaging (e.g., creatine-CEST), thereby revealing spatial heterogeneity and plasticity in conditions like obesity and aging.

However, several imaging constraints hinder clinical translation. Functional protocols require prolonged cold exposure (typically 1 - 2 h) to achieve sufficient metabolic activation for reliable detection. This requirement, combined with limited patient tolerance during extended scans, confines current applications primarily to research settings rather than routine clinical practice. Additionally, prolonged acquisitions require strategies to mitigate respiratory motion artifacts, particularly in supraclavicular regions. Emerging high-field systems (e.g., 5T or 7T MRI) hold promise for addressing this limitation through accelerated scanning. Besides, advanced MRI sequences, such as CEST and BOLD imaging, remain primarily research tools awaiting standardized clinical validation. Despite these limitations, MRI's non-invasive nature and lack of radiation exposure position it as a promising modality for clinical BAT imaging, offering distinct advantages and considerable potential for widespread implementation.

### CT

CT offers a pragmatic approach for extensive population-based BAT screening, leveraging its cost-effectiveness, rapid acquisition, and widespread availability. While radiation exposure remains the primary concern of CT imaging, low-dose non-enhanced chest CT scans have gained wide acceptance for lung cancer screening in the general population with high risk of lung cancers 107 and have been advocated for the primary prevention of cardiovascular disease 108. Importantly, chest CT scan can cover most BAT sites, including the supraclavicular region, armpits, mediastinum, and paraspinal region, enabling BAT assessment without any protocol modifications. This feature facilitates seamless integration of BAT detection into routine clinical practice, making chest CT a potential screening tool for the general population.

One previous study demonstrated that BAT exhibited slightly higher attenuation than WAT (-71.6 HU vs. -104.4 HU), which was attributable to its abundant mitochondria and reduced lipid droplets 22. Of active BAT, this difference became more pronounced due to increased blood flow and lipid consumption, resulting in further elevation of density 22. Ahmadi et al. identified -87 HU as the optimal cut-off CT value of activated BAT detection, with a sensitivity of 83.3% and specificity of 100% 109. However, distinguishing between BAT and WAT based solely on density poses a challenge due to their overlapping attenuation values, which complicates the accurate quantification of activated BAT volume. Additionally, BAT may be confused with inflammatory lesions, as both demonstrate increased density. Technical variability, like CT scan parameters and low-dose protocols, may influence fat adipose attenuation, warranting further investigation in the future. Despite these limitations, CT's widespread clinical use supports its role as a viable first-line tool for BAT surveillance in population studies.

### Infrared thermography

Infrared thermography (IRT), also known as infrared thermal imaging, represents a radiation-free, non-invasive approach for real-time BAT activity monitoring by detecting temperature changes in superficial depots. Previous studies have consistently shown an increase in temperature in the supraclavicular region following IRT stimulation with cold 110-113, supporting its potential as a population BAT screening tool. Law et al. reported an automatic overlap of 11.6% to 55.5% between IRT-derived thermal hotspots and PET-defined regions of maximal glucose uptake, indicating a correlation between thermographic signals and metabolic activity 114. However, IRT has several limitations: First, it is restricted to superficial coronal views of supraclavicular regions, precluding assessment of other planes and deeper depots. Second, temperature measurements are confounded by environmental factors and subject conditions, especially in superficial positions. Third, while thermal signals reflect BAT activation, the relationship between these signals and UCP1 expression levels remains unknown. Consequently, further studies are needed to solve the puzzle. Despite these limitations, IRT retains unique value in animal studies, where its non-invasive nature minimizes animal stress. Future work should establish standardized protocols to control for confounding variables and validate thermal signatures against molecular biomarkers, potentially through multimodal imaging approaches.

### Optoacoustic imaging

Optoacoustic imaging harnesses the photoacoustic effect to enable high-resolution visualization of light-absorbing tissue structures. When nanosecond laser pulses illuminate biological tissues, the absorbed light energy induces thermoelastic expansion, which in turn generates ultrasonic waves. These waves could be detected by ultrasound transducers, enabling real-time imaging of light-absorbing structures with high resolution and contrast 115. The modality of combining optical contrast with ultrasound resolution offers unique advantages for BAT assessment. Specifically, spectral optoacoustic imaging offers a remarkable capability to differentiate oxyhemoglobin and deoxyhemoglobin within the 700 - 900 nm near-infrared spectrum, enabling hemoglobin to function as an intrinsic contrast agent for monitoring metabolic activity via hemoglobin oxygenation dynamics 116. Notably, Reber et al. demonstrated the successful application of this technology in BAT imaging, effectively reflecting BAT activity through hemoglobin gradient analysis in both murine and human models. Furthermore, they established a complementary differentiation method based on the distinct optical signatures of lipid and water components within the 900 - 970 nm wavelength range 28. This technique offers several distinct advantages, including cost-effectiveness, user-friendliness, and absence of radiation or exogenous contrast agents, making it particularly suitable for repeated and longitudinal assessment of BAT in human studies. However, the restricted penetration depth (2-5 cm) and limited visual field, which are inherent defects of optoacoustic imaging, make it less likely to assess the total BAT mass and energy expenditure, especially in human subjects. Despite these limitations, optoacoustic imaging has significant potential for clinical translation. Its unique capability for real-time monitoring enables dynamic assessment of BAT metabolic activity *in vivo* under various physiological conditions, providing valuable insights into metabolic regulation mechanisms in living subjects.

### Xenon-enhanced imaging

Xenon is a lipophilic and inert gas. Its high atomic number and the significantly enhanced signal following hyperpolarization make it an excellent contrast agent for both CT and MR, with current applications in pulmonary function testing and cerebral perfusion imaging 117, 118. Xenon presents a strong affinity for BAT due to its lipophilic nature and the high blood perfusion of BAT. A series of animal studies, including mice and non-human primates, have shown that Xenon-enhanced CT could reflect BAT distribution, mass, and perfusion. More importantly, this technique remains unaffected by the host's metabolic state, which provides a suitable method of exploring the impact of metabolic disorders on BAT 29, 119. In active BAT, Xenon diffuses rapidly from the bloodstream into the extracellular space, reaching peak concentration within approximately 15 - 20 s and undergoing complete washout within 60 s 120. Such rapid metabolic process enables Xenon to visualize the perfusion of BAT dynamically; however, it also poses radiation concerns for CT.

Xenon-enhanced MRI can effectively address this limitation. Following inhalation of hyperpolarized ^129^Xe, rapid and repeated imaging can be performed using fast imaging sequences. Additionally, the chemical shift of Xenon dissolved in adipose tissue shows remarkable temperature sensitivity. By referencing its resonance frequency to methylene protons in triglycerides of adjacent BAT, both macro- and microscopic magnetic susceptibility gradient effects are eliminated, thereby enabling direct absolute temperature measurement and real-time monitoring in BAT 120, 121.

However, Xenon-enhanced imaging also has several limitations; the most notable limitation is the prohibitive cost of Xenon gas and the requirement of specialized equipment related to Xenon, including its supply and rebreathing systems. Besides, the inhaled Xenon concentration must be carefully maintained below 30% to avoid side effects, such as respiratory suppression 122. In the case of Xenon-enhanced MRI, the Xenon gas must be hyperpolarized before use. Moreover, since BAT is primarily distributed in the supraclavicular region, the imaging is highly sensitive to respiratory motion artifacts, thus requiring excellent breath control from the subject. Therefore, while this technique helps study BAT in research settings, the current limitations preclude its routine clinical adoption.

## AI

AI, including radiomics and deep learning, has revolutionized medical imaging and made significant inroads into the field of BAT research. The initial application of AI focused on BAT segmentation, while deep learning has demonstrated success in quantifying activated BAT volume in rodent models using MR sequences (e.g., Dixon, T2*, and fat fraction imaging) 123, 124. More recently, AI has been successfully applied to BAT detection, particularly in the analysis of CT imaging data. The predominance of CT-based AI models stems from two key factors: First, the PET-CT system provides intrinsically co-registered anatomical and metabolic data in a single scan, enabling precise BAT localization and feature analysis. Second, there are limited BAT-specific datasets from other imaging modalities, such as MR, optoacoustic imaging, and other PET imaging, which impede the development of equally robust models.

**Figure 5:**
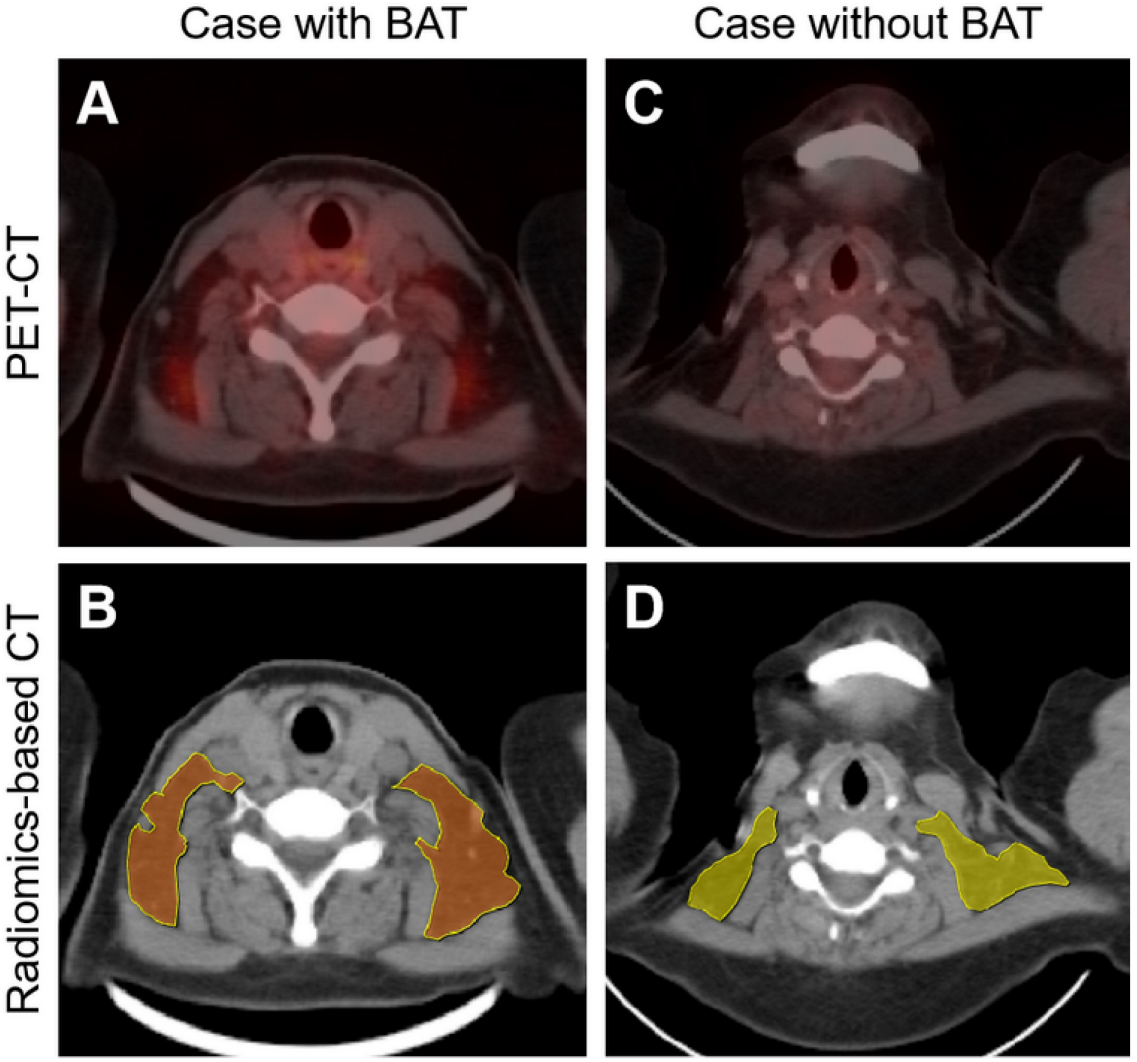
** Radiomics-based brown adipose tissue detection on CT.** PET-CT imaging (A and C) and radiomics-based CT imaging (B and D) of patients with or without brown adipose tissue (BAT). In the radiomics-based BAT detection model, BAT-positive and BAT-negative droplets are presented in brown (B) and yellow (D), respectively. Figure 5 is reproduced with permission from Ivyspring International Publisher (Theranostics. 2023; 13: 1584-93), an open access article under a CC-BY 4.0 license. BAT: brown adipose tissue; CT: computed tomography; PET: positron emission tomography.

Initial breakthroughs were made via radiomics-based analysis. Radiomics not only detects subtle and invisible differences in images but also analyzes and summarizes these differences, enabling radiologists to identify lesions that are difficult to detect 125. Nazeri et al. extracted and analyzed the radiomics features of BAT from PET-CT images collected from 18 volunteers. In total, there were 66 high-definition PET (HD PET), 66 non-HD PET, and 6 CT features that showed high-repeatable radiomic features, validating the feasibility of detecting BAT on PET and CT using radiomics 126. Li et al. successfully developed the radiomics-based BAT detection model, achieving 68.8% accuracy, 78.8% sensitivity, and 58.7% specificity, with an area under the curve (AUC) of 0.85. The model was trained using the BAT target delineation on non-enhanced CT images, which were referred to as PET images (**Figure 5**) 127. However, the efficacy of the radiomics model should be improved, particularly in terms of specificity. Additionally, the adipose droplet needed to be outlined manually, a laborious and time-consuming process that limited its clinical application.

Deep learning approaches provide a superior solution for BAT detection, demonstrating higher efficacy and eliminating the need for manual segmentation. Erdil et al. developed CT-based BAT prediction models using convolutional neural networks to predict FDG uptake in supraclavicular adipose tissue. Notably, the models trained on cold-exposure cohorts showed moderate performance, with a Dice score ranging from 0.521 to 0.745, but exhibited limited generalizability when tested in retrospective, non-cold-exposure cohorts 128. While these studies have demonstrated the feasibility of BAT detection on CT using AI, the models are still far from being ready for routine clinical application. Current models rely heavily on subtle characteristics, such as texture and histogram features, which are greatly influenced by acquisition parameters 125, 129, 130. Future efforts should focus on standardizing scanning parameters and improving model generalization across diverse patient populations and scanning conditions.

Although AI offers a promising approach for BAT detection on CT images, developing an accurate BAT detection model also presents challenges. The limited availability of PET-CT data and the relatively recent standardization of BAT imaging criteria (2016) 70 have contributed to a shortage of high-quality training data for developing BAT AI models. To date, the most extensive BAT study analyzed approximately 1,300,000 PET-CT scans (5.9% BAT positive) 61, but relied solely on imaging reports rather than raw images, which were unable to provide imaging data. Most contemporary BAT imaging studies enroll only 10 to 500 participants, which could hardly satisfy the requirement for AI model development, especially for deep learning models. Moreover, retrospective [^18^F] FDG PET-CT imaging data, the primary training source, present inherent limitations: Within retrospective images without cold exposure, BAT is usually found out by chance, which may be activated by various factors (cold exposure, drug stimulation, and disease), introducing uncontrolled variability.

Furthermore, inactive BAT shows no FDG uptake, which may result in misclassification as BAT-negative samples and confusion with BAT-absent samples, thereby introducing uncertainty into training datasets. Prospective cold-exposure cohorts (e.g., Activating Brown Adipose Tissue Through Exercise (ACTIBATE) cohort 131 and the Basel cohort 132) address some limitations by performing the standard cold exposure process before the PET-CT scan. Such imaging data, controlled for confounding factors and activated BAT as fully as possible, were ideal for model development. Nevertheless, the cohort data had age-related bias, as they enrolled participants aged 18 to 26 years to ensure the positive rate of BAT. Besides, models trained on such controlled data exhibited poor performance in retrospective datasets, indicating limited generalization 128. Above all, a dual-path solution may be optimal combining large retrospective datasets (for sample size) with controlled interventional data (for quality) for model training, supplemented by histological validation to ensure biological relevance. This approach would leverage the sample size advantages of retrospective data while improving model efficacy and generalization through controlled studies. Ultimately, such integration could bridge the gap between research and clinical application of AI in BAT detection.

## Future perspectives

Initially, studies focused primarily on BAT's thermogenic properties to dissipate energy as heat. Subsequent evidence has revealed its broader metabolic roles, including enhancing insulin sensitivity and lipid metabolism, resulting in systemic metabolic homeostasis 4, 5, 7. This growing body of research highlights the significance of BAT in maintaining energy balance and its potential implications for combating metabolic disorders, spurring exploration of BAT activation and transplantation strategies for obesity, diabetes, and other related conditions management 47, 133. However, while clinical interest in BAT has surged, current detection methods remain inadequate: [^18^F] FDG PET-CT as the gold standard for detecting BAT, requires prolonged protocols (≥60 min rest for radiotracer distribution) and pharmacological exposure (typically 1 - 2 h) to achieve sufficient BAT activation, limiting its widespread clinical adoption. However, the unique ability of PET to quantify activated BAT remains indispensable for investigating therapeutic applications, including cardiometabolic disease interventions, weight-loss strategies, immune modulation, and cancer cachexia management.

Given the urgency of developing effective detection methods, there is a clear need for novel approaches that can provide safe and rapid assessments of BAT. While advances in imaging technologies have shown promise, critical methodological gaps persist, particularly the absence of standardized diagnostic criteria. To date, only [^18^F] FDG PET-CT has validated diagnostic criteria for BAT in imaging (BARCIST 1.0) 70, whereas novel method studies remain in the feasibility stage without standardized protocols or robust diagnostic criteria. Furthermore, due to insufficient sample sizes and population bias, some diagnostic criteria are not robust and universal enough. Therefore, both optimal BAT imaging protocols and precise BAT definition are necessary, which are also complementary to each other.

The clinical relevance of distinguishing BAT from beige adipose tissue is debatable. Today, no existing imaging modality can reliably separate BAT and beige adipose tissue due to their overlapping anatomy (both prevalent in the cervical and supraclavicular regions) and similar function 9, 30. The key differences between them primarily involve their cellular characteristics and molecular markers. Beige adipocytes are transformed from white adipocytes under stimulation, and they return to white adipocytes after the stimulus is withdrawn 30. However, current imaging modalities failed to distinguish this beigeing process from BAT activation in imaging. Additionally, beige adipocyte expresses unique markers, such as PAT2 and P2RX5 9. It is unclear whether the difference in the markers would lead to various endocrine functions. Further studies could be conducted to investigate the differences in markers between BAT and beige adipose tissues, clarifying whether there are any subtle functional differences.

As research continues to unfold, translational progress will require deeper collaboration between basic medicine and clinical applications. Bridging mechanistic insights with methodological innovation can accelerate the development of an effective, safe, and convenient BAT detection method, with great generalization and practicality. The ongoing exploration and imaging of BAT have the potential to reshape our understanding of metabolism and provide new strategies for managing related conditions.

## Conclusions

Initially characterized as a thermogenic organ, BAT is now the focus of intense investigation owing to its significant endocrine capabilities. Mirroring its growing clinical relevance, the development of BAT detection methodologies has expanded rapidly. Although [^18^F] FDG PET-CT has limited applicability in general population studies, it remains the diagnostic gold standard due to mature protocols and standardized imaging criteria. Emerging techniques, such as MRI and optoacoustic imaging, show significant potential as next-generation longitudinal monitoring tools due to their non-invasive and radiation-free properties. Meanwhile, AI-powered analysis of non-contrast CT scans could enable large-scale population screening. These modalities possess complementary strengths; maximizing their respective advantages is essential for both elucidating fundamental mechanisms of BAT and advancing clinical translation. Nevertheless, standardized protocols and comprehensive clinical validation remain imperative for these novel approaches, while the accuracy and convenience of these methods are the main factors that need to be considered. Future studies should focus on establishing stable and robust BAT imaging characteristics, which are supported by a reliable mechanism, while optimizing and standardizing imaging protocols to enhance seamless clinical integration and technology translation.

## Figures and Tables

**Table 1:** Summary of imaging methods in brown adipose tissue detection

Method	Imaging target	Advantages	Limitations	References
PET				
[^18^F] FDG	Glucose	Ÿ Standardized imaging workflow Ÿ Well-established method for BAT imaging that enables critical research into therapeutic applications	Ÿ Prolonged preparation and imaging procedures with radiation exposure Ÿ Affected by metabolic disorders Ÿ Dissociated with the activity of BAT	17-19, 69-71, 74
[^18^F] FTHA	Fatty acid	Ÿ Not affected by metabolic disorders Ÿ Well reflects the active volume of BAT	Ÿ Prolonged preparation and imaging procedures with radiation exposure Ÿ Slow uptake rate and non-specific uptake	20, 73, 74, 76-78
[^11^C] palmitate	Fatty acid	Ÿ Not affected by metabolic disorders Ÿ Well reflects the active volume of BAT	Ÿ Prolonged preparation and imaging procedures with radiation exposure Ÿ Slow uptake rate and non-specific uptake Ÿ Short half-life (20 min)	20, 73, 74, 76
[^18^F] AraG	Arabinosyl guanine analog	Ÿ Reflects both BAT and immune state simultaneously Ÿ A promising way for exploring the relationship between BAT and cancer	Ÿ Prolonged preparation and imaging procedures with radiation exposure Ÿ Preliminary stage of exploration	23, 81-84, 134
[^18^F] FM-PBR28, [^18^F] FEPPA and [^18^F] FDPA	Translocator protein	Ÿ Activity-independent, captures both active and inactive BAT depots, provides a complete anatomical distribution map Ÿ Detects newly formed beige fat without stimulation	Ÿ Limited in the evaluation of BAT functional state Ÿ Primary validation in animal studies	24, 86-89
**SPECT**	Targets according to traces, like sympathetic nerve ([^123^I] MIBG), fatty acid ([^123/125^I] BMIPP), and tissue perfusion ([^99m^Tc] MIBI)	Ÿ Lower radiation exposure and cost compared to PET Ÿ Diverse tracer applications	Ÿ Prolonged preparation and imaging procedures Ÿ Lower resolution and sensitivity, with long acquisition time Ÿ Depth-dependent photon attenuation and uncontrollable factors (dead-time/spill-over effects) hinder accurate quantification	79, 90-92
**MRI**				
Dixon	Water-fat ratio	Ÿ Non-radiation exposure Ÿ Reflects the metabolic activity of BAT	Ÿ Affected by magnetic field inhomogeneity artifacts, and phase wrapping Ÿ Indistinct cut-off value between BAT and WAT	21, 95-98,
T2* weighted imaging	T2* value	Ÿ Non-radiation exposure Ÿ Reflects the metabolic activity of BAT	Ÿ Affected by blood perfusion and local tissue temperature	25, 99-101
Creatine-CEST	Concentration of creatine	Ÿ Non-radiation exposure Ÿ Sensitive to the activity of BAT, reflects BAT activity at room temperature	Ÿ Unclear definition of BAT Ÿ Influenced by microenvironment, such as temperature, pH, and direct water saturation	38, 102
BOLD	Oxygen consumption and blood flow	Ÿ Non-radiation exposure Ÿ Well reflects the metabolic activity of BAT	Ÿ Affected by blood perfusion and local-tissue temperature	27, 106
**CT**	Density	Ÿ Easy to acquire and measure Ÿ Cost-efficient and generalizable for BAT screening	Ÿ Radiation exposure Ÿ Indistinct cut-off value between BAT and WAT Ÿ Easily confused with inflammation	22, 109
**IRT**	Temperature	Ÿ Non-radiation exposure Ÿ Easy to acquire and measure	Ÿ Limited planes and BAT sites Ÿ Unclear correlation between temperature and BAT activity	110-112, 114
**Optoacoustic imaging**	Light-absorbing structures, such as, oxyhemoglobin, deoxyhemoglobin, lipid, and water	Ÿ High-resolution and real-time dynamic detection, without radiation exposure Ÿ Enable BAT activity assessment with an intrinsic contrast agent	Ÿ Restricted penetration depth (2 - 5 cm) and limited visual field	28, 115, 116
**Xenon-enhanced imaging**	Lipid and blood perfusion	Ÿ Strong affinity for BAT due to its lipophilic nature and high blood perfusion that allows clear visualization of BAT distribution, mass, and blood flow Ÿ Allows absolute temperature measurement in BAT	Ÿ High operational cost for Xenon gas and specialized equipment (Xenon gas supply / rebreathing systems) Ÿ Safety constraints - Inhaled xenon must be kept below 30% concentration to prevent respiratory depression	29, 119-122
**AI**	Subtle and tiny differences captured and summarized by radiomics or deep learning	Ÿ Cost-efficient and generalizable for BAT screening	Ÿ Unsatisfactory efficacy and low generalization of the model Ÿ Acquiring sufficient and high-quality data for model training	126-128

AI: artificial intelligence; AraG: arabinosyl guanine analog; BAT: brown adipose tissue; BOLD: blood oxygenation level-dependent; CT: computed tomography; CEST: chemical exchange saturation transfer; FDG: fluorodeoxyglucose; [^18^F] FTHA: 14(R,S)-[^18^F] Fluoro-6-thia-heptadecanoic acid; [^18^F] FM-PBR28: N-(2-(2-[^18^F] Fluoroethoxy) benzyl)-N-(4-phenoxypyridin-3-yl) acetamide; [^18^F] FEPPA: N-[2-[2-[^18^F] Fluoroethoxy] benzyl]-N-(4-phenoxypyridin-3-yl) acetamide; [^18^F] FDPA: 7-(2-[^18^F] Fluoroethyl)-6,8-dihydro-5H-pyrido [2,3-b] indole; IRT: infrared thermography; [^123^I] MIBG: [^123^I] metaiodobenzylguanidine; [^123/125^I] BMIPP: [^123/125^I] β-Methyl-*ρ*-iodophenyl-pentadecanoic acid; SPECT: single photon emission computed tomography

## Abbreviations

BAT: brown adipose tissue
MOVA: 3-methyl-2-oxovaleric acid
BHIBA: β-hydroxyisobutyric acid
12,13-diHOME: 12,13-dihydroxy-9Z-octadecenoic acid
FGF21: fibroblast growth factor 21
[^18^F] FDG: ^18^F-fluorodeoxyglucose
PET: positron emission tomography
AI: artificial intelligence
SPECT: single photon emission computed tomography
MRI: magnetic resonance imaging
CT: computed tomography
IRT: infrared thermography
[^18^F] AraG: [^18^F] arabinosyl guanine analog
CEST: chemical exchange saturation transfer
WAT: white adipose tissue
BOLD: blood oxygenation level-dependent
Myf5: myogenic transcription factor 5
UCP1: uncoupling protein 1
TNAP: tissue-nonspecific alkaline phosphatase
SERCA: sarcoendoplasmic reticulum calcium ATPase
ER: endoplasmic reticulum
RYR: ryanodine receptor
BARCIST: Brown Adipose Reporting Criteria in Imaging STudies
HU: Hounsfield unit
[^18^F] FTHA: 14(R,S)-[^18^F] Fluoro-6-thia-heptadecanoic acid
[^123/125^I] BMIPP: [^123/125^I] β-methyl-ρ-iodophenyl-pentadecanoic acid
TSPO: translocator protein
[^18^F] FM-PBR28: N-(2-(2-[^18^F] Fluoroethoxy) benzyl)-N-(4-phenoxypyridin-3-yl) acetamide
[^18^F] FEPPA: N-[2-[2-[^18^F] Fluoroethoxy] benzyl]-N-(4-phenoxypyridin-3-yl) acetamide
[^18^F] FDPA: 7-(2-[^18^F] Fluoroethyl)-6,8-dihydro-5H-pyrido [2,3-b] indole
[^123^I] MIBG: [^123^I] metaiodobenzylguanidine
[^99m^Tc] MIBI: [^99m^Tc] methoxyisobutylisonitrile
IDEAL: iterative decomposition of water and fat with echo asymmetry and least-squares estimation
HD PET: high definition PET
AUC: area under curve
ACTIBATE: Activating Brown Adipose Tissue Through Exercise
